# Low levels of dehydroepiandrosterone sulfate are associated with the risk of developing cardiac autonomic dysfunction in elderly subjects

**DOI:** 10.20945/2359-3997000000104

**Published:** 2019-02-01

**Authors:** Marina de Paiva Lemos, Munique Tostes Miranda, Moacir Marocolo, Elisabete Aparecida Mantovani Rodrigues de Resende, Rosângela Soares Chriguer, Carla Cristina de Sordi, Octávio Barbosa

**Affiliations:** 1 Universidade Federal do Triângulo Mineiro Universidade Federal do Triângulo Mineiro Departamento de Ciências do Esporte Uberaba MG Brasil Departamento de Ciências do Esporte, Universidade Federal do Triângulo Mineiro (UFTM), Uberaba, MG, Brasil; 2 Universidade Federal de Juiz de Fora Universidade Federal de Juiz de Fora Departamento de Fisiologia Juiz de Fora MG Brasil Departamento de Fisiologia, Universidade Federal de Juiz de Fora (UFJF), Juiz de Fora, MG, Brasil; 3 Universidade Federal do Triângulo Mineiro Universidade Federal do Triângulo Mineiro Departamento de Endocrinologia e Metabolismo Uberaba MG Brasil Departamento de Endocrinologia e Metabolismo, Curso de Pós-Graduação em Ciências da Saúde, Universidade Federal do Triângulo Mineiro (UFTM), Uberaba, MG, Brasil; 4 Universidade Federal de São Paulo Universidade Federal de São Paulo Departamento de Biociências Santos SP Brasil Departamento de Biociências, Universidade Federal de São Paulo (Unifesp), Santos, SP, Brasil

**Keywords:** Aging, cardiac autonomic modulation, cardiovascular disease, dehydroepiandrosterone sulfate, heart rate variability

## Abstract

**Objective:**

To assess the relationships between serum dehydroepiandrosterone sulfate (DHEA-S) levels and heart rate variability (HRV) among different age groups.

**Subjects and methods:**

Forty-five healthy men were divided into 3 groups: young age (YA; 20-39 yrs; n = 15), middle age (MA; 40-59 yrs; n = 15) and old age (OA; ≥ 60 yrs; n = 15). Hemodynamic parameters, linear analyses of HRV and concentrations of cortisol and DHEA-S were measured at rest.

**Results:**

The OA group presented a higher resting heart rate (84.3 ± 4.6 bpm) than the YA group (72.0 ± 4.4 bpm; p < 0.05). The YA group showed an attenuated variance of HRV (2235.1 ± 417.9 ms^2^) compared to the MA (1014.3 ± 265.2 ms^2^; p < 0.05) and OA (896.3 ± 274.1 ms^2^; p < 0.05) groups, respectively. The parasympathetic modulation of HRV was lower in both the MA (244.2 ± 58.0 ms^2^) and OA (172.8 ± 37.9 ms^2^) groups in comparison with the YA group (996.0 ± 255.4 ms^2^; p < 0.05), while serum DHEA-S levels were significantly lower in both the MA (91.2 ± 19.6 mg/dL) and OA (54.2 ± 17.7 mg/dL) groups compared to the YA group (240.0 ± 50.8 mg/dL; p < 0.05). A positive correlation between lower serum concentrations of DHEA-S and attenuated variance of HRV (r = 0.47, p = 0.031), as well as lower serum concentrations of DHEA-S and decreased parasympathetic modulation of HRV (r = 0.54, p = 0.010), were found.

**Conclusion:**

The present study demonstrated that the decline of plasma DHEA-S is associated with reduced cardiac autonomic modulation during the aging process.

## INTRODUCTION

Protective steroid hormones decrease with aging, particularly dehydroepiandrosterone (DHEA) and its active metabolite dehydroepiandrosterone sulfate (DHEA-S). These two endogenous hormones are synthesized from cholesterol and excreted mainly by the reticular zone of the adrenal cortex; they represent the most abundant steroid hormones in circulation across the life span (1,2). Recent scientific and public interest in DHEA-S has been driven in large part by the evidence frequently published in recent years, suggesting a strong relationship between the decline of the endogenous hormone and aging (3).

An excessive amount of mineralocorticoid hormones has been linked to cardiovascular disorders such as cardiac hypertrophy, arrhythmias, endothelial and smooth muscle vascular dysfunctions. Previous studies showed a causal association between steroid hormones and cardiovascular diseases (4). A large majority of research has hypothesized that the development of DHEA-S deficiencies in elderly individuals might play a substantial role in the damage of many functions and contribute to the development of several injuries, including cardiovascular impairment (5), regardless of other risk factors (6). Moreover, it has been suggested that low serum levels of DHEA-S are a predictor of cardiovascular disease mortality (7).

A substantial number of investigations have focused on biomarkers that identify subjects at higher risk of developing cardiovascular disease (8). One important indicator that seems to play a determinative role is vagal and sympathetic cardiac autonomic dysfunction. In fact, increased sympathetic and/or decreased parasympathetic activity has been associated with increased risk for cardiovascular disorders such as hypertension, heart failure, ventricular arrhythmias and, especially, sudden cardiac death (9). Measurement of heart rate variability (HRV) is a valid noninvasive technique for estimating the characteristics of the autonomic nervous system and quantifying modulation of the sympathetic and parasympathetic inputs (10). HRV is also believed to decline as people age, but an important scientific question is whether decreases in HRV occur naturally with age as a result of the aging process itself or as a result of pathogenic processes. Therefore, the relationship between serum levels of sex steroid hormones and autonomic functions in both sexes has become an attractive area of research.

Scientific evidence has shown that elements such as aging and levels of sex hormones may also influence cardiovascular autonomic control (11,12). Data obtained by our group demonstrated that DHEA-S decline was associated with lower HRV in sedentary elderly individuals compared to physically active individuals (data not shown). Nevertheless, few marked results have been clearly reported in relation to serum DHEA-S levels and HRV in the aging process. Therefore, it is believed that lower blood concentrations of DHEA-S along with the advancement of age can interfere in the circadian oscillation of the autonomic system and thus unbalance the sympathetic-vagal activity and cardiovascular function, consequently increasing the risk of cardiovascular diseases in this population.

Therefore, in the present report, we examine the serum DHEA-S levels and cardiac autonomic control using linear methods of HRV in subjects in different age groups. Our hypothesis was that DHEA-S decline would be most pronounced elderly individuals over 60 years old and that this reduction could be associated with cardiac autonomic dysfunction.

## SUBJECTS AND METHODS

### Study population

Forty-five healthy men without severe complications participated voluntarily in this study and were divided into 3 groups according to age: young-man group (n = 15), composed of people between the ages of 20 and 39 years; middle-age group (n = 15), composed of people between the ages of 40 and 59 years and old-man group (n = 15), composed of people aged 60 years and over. Exclusion criteria included coronary artery disease, valvular and congenital heart disease, congestive heart failure, diabetes mellitus, sinus tachycardia, smoking habit and psychiatric, respiratory or metabolic disorders. All subjects gave written informed consent, and the study was approved by the Ethical Committee for Research of the Federal University of Triângulo Mineiro (Protocol: 2580/2013).

### Body composition and anthropometric measures

Anthropometric measurements of weight, height and body mass index were taken using a portable Tanita electronic scale (Tanita HD-350^®^) and portable stadiometer (0.1 cm precision; Prime Med^®^) fixed on a wall with volunteers wearing no shoes and light clothes. The percentage of body fat and the resulting fat-free body mass were estimated using the three-skinfold method.

### Experimental protocol

Data collection was done from 8:00 to 10:00 a.m., 2 h after the regular first breakfast. All participants underwent anamnesis and were instructed to fast and not consume alcohol or caffeine products. Additionally, they were told not to exercise on the day of the experiment. The experimental sessions were performed in baseline condition (supine position). After 10 min of resting in a quiet, temperature-regulated room, the electrocardiographic signal was recorded for 5 min. Subjects were not allowed to sleep. The electrocardiographic signal was continuously amplified by an electrocardiogram (ECG) recorder (model ECG-5, EcafixFunbec^®^, São Paulo, Brazil), collected by an analogue to digital converter board (DI-194 Starter Kit, Dataq Inst., Akron, OH, USA) with a sampling rate of 240 Hz and stored on personal computer for posterior offline analysis. Blood pressure (BP) was monitored noninvasively using an automatic and oscillometric cuff (M3 Intellisense HEM-7051-E; Omron Healthcare^®^, Kyoto, Japan). The HR was monitored through lead II of an ECG (model ECG-5, EcafixFunbec^®^, São Paulo, Brazil) beat per beat.

### Heart rate variability measurements

The HRV was assessed in the time domain by means of time series variance. Subsequently, frequency domain analysis was performed with an autoregressive algorithm (13). In brief, a time series of the RR intervals was calculated via the Levinson-Durbin recursion, with the order of the model chosen according to Akaike's criterion. The temporal indices analyzed were: RR intervals (iRR, ms) and variance. The variance was estimated as a marker of total variability in the time domain. The power spectral density was calculated for each RR series. Spectral components were considered: low frequency (LF: 0.04 to 0.15 Hz) and high frequency (HF: 0.15 to 0.40 Hz). The spectral components were expressed in absolute (ms^2^) and normalized units (nu). In addition, the LF/HF-ratio was calculated, a sensitive indicator for sympathetic activation. Normalization consisted of dividing the power of a given spectral component by the total power minus the power below 0.03 Hz and multiplying the ratio by 100 (13).

### Hormonal analysis

At the end of the protocol, blood samples were drawn from each participant through antecubital vein puncture of the right arm to evaluate serum hormone levels of cortisol and DHEA-S. For collection, BD Vacutainer^®^ SST II Advance tubes were used to obtain and separate a serum hormone sample. These tubes were centrifuged for 5 min at 1000 rpm. Next, the serum in test tubes was stored at −80 °C for further analysis. The intra- and inter-assay coefficients of variation were below 8% for DHEA-S and cortisol, respectively, and were measured using the direct chemiluminescent assay method (Advia Centaur, Bayer Corporation, Tarrytown, NY, USA).

### Statistical analyses

Data are expressed as mean ± standard error of the mean (SEM). The normality of the data distribution was verified by the Shapiro-Wilk test, and Levene's test was used to evaluate the homogeneity of the sample. The variables were compared among the 3 groups using a 1-way analysis of variance (ANOVA). When overall differences were found at p < 0.05, a post hoc Tukey's test protected least-significant difference was performed, or Kruskal-Wallis, followed by Dunn's post hoc test in agreement with presence or not of distribution normality and/or homogeneity of the variance, respectively. The Pearson correlation coefficient was used to test the correlation between HRV and serum DHEA-S concentrations. The level of significance was set at p ≥ 0.05 for all analyses. Analyses were conducted using SigmaStat (Jandel Scientific Software; SPSS, Chicago, IL).

## RESULTS

Information regarding age, body composition and anthropometric parameter recordings according to age group is shown in Table 1. The old-man group presented a significantly higher age in comparison to the middle-age and young-man groups (p < 0.05), and participants in the middle-age group were significantly older than those in the young-man group (p < 0.05). There were no significant differences between the three groups in terms of weight, BMI, muscle mass and total body fat (p > 0.05).

**Table 1:** Body composition and anthropometric characteristics

	YA (20-39 years)	MA (40-59 years)	OA (≥ 60 years)
Age (y)	29.2 ± 3.6	52.9 ± 4.5*	62.8 ± 2.4* †
Weight (kg)	63.8 ± 11.2	63.9 ± 11.0	65.2 ± 13.6
Height (cm)	163.5 ± 4.6	161.8 ± 7.9	160.9 ± 11.4
BMI (kg/m^2^)	23.9 ± 5.1	24.4 ± 8.9	25.1 ± 7.3
Total fat (kg)	16.2 ± 3.0	17.2 ± 2.8	19.3 ± 2.3
Total MM (kg)	47.6 ± 5.2	46.7 ± 8.2	45.9 ± 11.3

Values are expressed as mean ± standard error (SE). BMI: body mass index; MM: muscle mass; YA: young-age; MA: middle-age; OA: old-age; respectively.

* p < 0.05 *vs*. YA and

† p < 0.05 *vs*. MA.

P values for comparisons between groups using one-way ANOVA followed by Tukey's post-hoc analysis.

**Figure 1:**
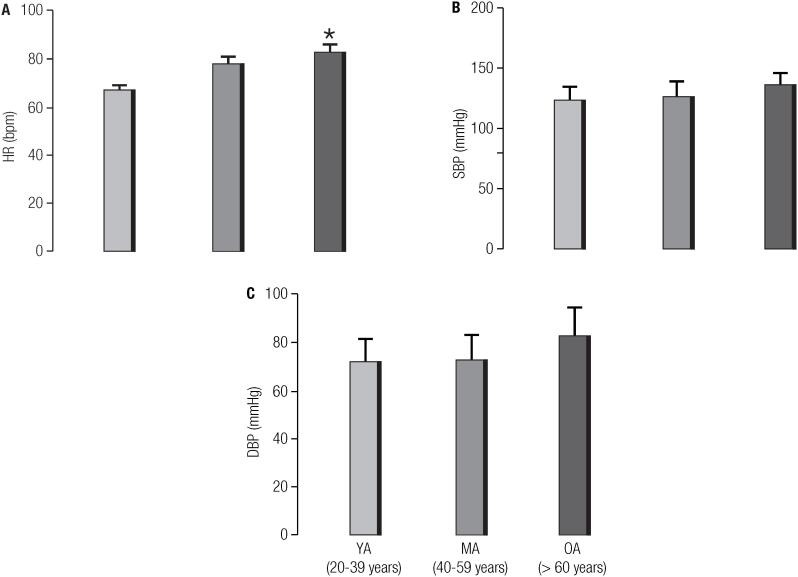
Graphs showing hemodynamic parameters in young-man, middle-age and old-man. (A) heart rate, (B) systolic blood pressure and (C) diastolic blood pressure at rest. Data are mean ± standard error (SE). *p < 0.05 vs. young-man. P values for comparisons between groups using one-way ANOVA followed by Tukey's post-hoc analysis or Kruskal-Wallis followed by Dunn's post hoc test.

Resting HR and BP are shown in Figure 1. The HR of elderly participants aged over 60 years at rest was higher (82.8 ± 3.2 bpm) than that of the young-man group (67.0 ± 2.1 bpm; p < 0.001) (Figure 1A). In contrast, there were no significant differences between groups in terms of systolic BP (123.5 ± 11.3 mmHg in young-man group, 126.5 ± 12.0 mmHg in middle-age group and 136.4 ± 10.4 mmHg in old-man group; Figure 1B) and diastolic BP (71.8 ± 9.5 mmHg in young-man group, 72.6 ± 10.1 mmHg in middle-age group and 82.4 ± 11.9 mmHg in old-man group; p > 0.05) (Figure 1C).

Table 2 shows the HRV by linear analysis. Compared to the young-man group, the middle-age and old-man groups presented an attenuated variance of HRV (p < 0.001). The HF oscillations of HRV were lower in both the middle-age and old-man groups compared to the young-man subjects (p < 0.001). The LF/HF ratio was lower in the young-man group than in the middle-age and old-man groups (p < 0.05). No significant changes in sympathetic indices of HRV were found (p > 0.05).

**Table 2:** Spectral parameters of heart rate calculated from time series using autoregressive power spectral analysis at rest in young-man, middle-age and old-man

	YA (20-39 years)	MA (40-59 years)	OA (> 60 years)
Variance, ms^2^	2860.5 ± 452.7	1034.2 ± 146.6*	702.7 ± 177.6*
LF, ms^2^	465.6 ± 78.6	415.4 ± 32.7	433.7 ± 154.3
LF, nu	41.2 ± 7.4	52.6 ± 1.7	48.0 ± 6.2
HF, ms^2^	1004.2 ± 112.8	266.3 ± 36.8*	179.5 ± 36.2*
HF, nu	66.8 ± 8.1	53.5 ± 4.3	47.3 ± 4.3
LF/HF ratio	0.82 ± 0.3	1.66 ± 0.2*	2.20 ± 0.4*

Values are expressed as mean ± standard error (SE). LF: low-frequency spectral component of HRV; HF: high-frequency spectral component of HRV; nu: normalized units.

* p < 0.05 *vs*. YA.

P values for comparisons between groups using one-way ANOVA followed by Tukey's post-hoc analysis or Kruskal-Wallis followed by Dunn's post hoc test.

**Figure 2:**
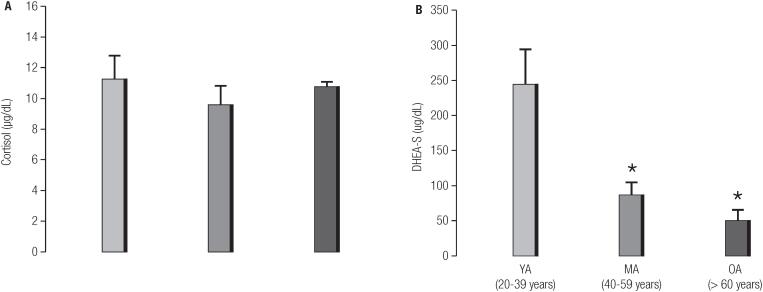
Bar graphs (mean ± SE) showing serum concentrations of (A) cortisol and (B) DHEA-S among young-man, middle-age and old-man. *p < 0.05 vs. young-man. P values for comparisons between groups using one-way ANOVA followed by Tukey's post-hoc analysis or Kruskal-Wallis followed by Dunn's post hoc test.

Figure 2 presents the serum levels of cortisol and DHEA-S in all groups. As shown in the figure, no statistically significant difference was observed between the three groups in terms of cortisol parameters (11.2 ± 1.6 mg/dL in young-man group; 9.6 ± 1.2 mg/dL in middle-age group and 10.7 ± 0.4 mg/dL in old-man group; p > 0.05) (Figure 2A). On the other hand, the serum levels of DHEA-S were significantly lower in both the middle-age (87.4 17.8 mg/dL) and old-man (50.8 ± 14.9 mg/dL) groups compared to the young-man group (244.3 ± 49.5 mg/dL; p < 0.005) (Figure 2B).

**Figure 3:**
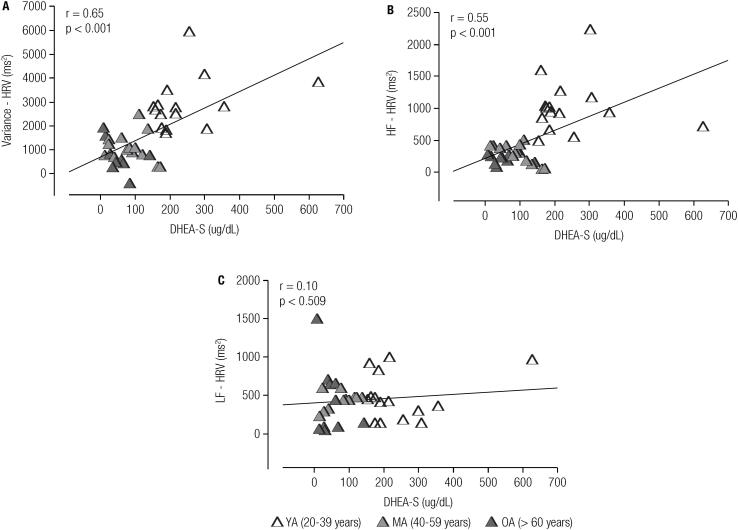
Pearson's linear correlation scatter plot between serum DHEA-S levels and variance of HRV (A), serum DHEA-S levels and high-frequency spectral component of HRV (B) and serum DHEA-S levels and low-frequency spectral component of HRV (C).

Analysis showed a significant and positive correlation between serum concentrations of DHEA-S and variance of HRV (r = 0.65, p < 0.001; Figure 3A), as well as a significant correlation between serum concentrations of DHEA-S and absolute HF oscillations of HRV (r = 0.55, p < 0.001; Figure 3B). There were no significant correlations between serum concentrations of DHEA-S and the LF component of HRV (r = 0.10, p = 0.509; Figure 3C).

## DISCUSSION

Studies have indicated that decreased levels of DHEA-S have been found in people with cardiovascular disease (14), and an inverse correlation between DHEA-S and cardiovascular mortality has been reported in the aging process (15). On the other hand, researchers have documented that HRV decreased with aging independent of pathological conditions or medication use (16), potentially suggesting that cardiac autonomic modulation diminishes due to normative aging and HRV changes due to chronic diseases or health-related conditions (17). Theoretically, therefore, the main finding of this large study was that a decline of serum DHEA-S level is positively correlated with lower HRV and parasympathetic modulation and negatively correlated with sympathetic activities. An important factor should be taken into account: The HRV normally decreases with age, as well as serum DHEA-S levels; therefore, there is a conflict to understand if the correlation between DHEA-S and HRV found in our study is causal or noncausal. Previous studies have demonstrated a decrease in DHEA-S concentration with aging, and this decrease is associated with greater cardiac autonomic dysfunction; other studies indicate that there is no correlation between these variables because both decline physiologically due to aging. In the current scientific literature, there are limited studies on the association between cardiac function and serum levels of DHEA-S.

The effect of age on DHEA-S levels in adults has previously been described (18). Our study confirms the results of these earlier findings and clearly demonstrates that serum DHEA-S levels significantly decline with age. It is worth noting that within an individual, the levels of DHEA-S may fluctuate over time, but the general trend is a decline (19). The serum DHEA and DHEA-S have been deeply studied for their potential anti-aging effects. In humans, blood DHEA levels decline with age, suggesting that it may be a key element in the aging process (20). Studies in mice have shown that DHEA-S supplementation reverted left ventricular rigidity in aging (11). However, it is important to emphasize that the American Society of Endocrinology does not recommend the supplementation of DHEA-S for normal subjects, even during aging. Another study demonstrated an inverse relationship between heart disease and serum DHEA-S levels (21). Prior studies have shown associations between sex steroid levels, age and autonomic activity. In fact, testosterone effects on autonomic control of nerve transduction pathways have been described, providing evidence that this hormone affects parasympathetic responses and facilitates the baroreflex sensibility in autonomic function. Although it is not an adrenal hormone, growth hormone (GH) levels in circulation begin to decline soon after attainment of adult body size and full physical and reproductive maturation. GH deficiency is associated with increased cardiovascular morbidity and mortality. Abnormalities in HRV have been found in patients with GH deficiency (22–24); however, these alterations are only associated with decreased cardiac sympathetic activity (25). These results indicate that serum steroid hormones have effects not only on sexual differentiation but also on autonomic modulation of the cardiovascular system. To our knowledge, so far, there is very little published evidence regarding the effects of aging on DHEA-S concentrations and their association with the HRV. Studying the relationship between serum sex steroid levels and HRV parameters, Doĝru and cols. found that DHEA-S exhibited the most significant correlations with autonomic functions after controlling for the effects of age in comparison to gonadal steroids (12).

In part, our results may be explained by the existence of cardiac receptors for DHEA-S verified in some studies (11). On the other hand, it was shown that DHEA-S had regulatory effects on sympathetic adrenal activity and was positively correlated with the attenuation of the sympathetic-adrenal response to hypoglycemia. The mechanisms of the effects of DHEA-S on the sympatho-vagal balance have not yet been elucidated; therefore, this action can be attributed to specific receptors and the effect of DHEA-S on the brain (26).

DHEA-S has been considered a marker of good health (27). The peak of this hormone proceeds to attenuate with age more markedly than any other endogenous hormone, corroborating the experimental evidence of their involvement in age-related health disorders (19). Interaction of the DHEA-S blood levels and aging-related pathologies has been widely explored during the last decades. Several researches have shown a negative association between the levels of these hormones and the aging process, as defined by the occurrence of numerous disorders including cardiovascular disorders, as well as more definitive entities such as mortality (28). The literature reported that DHEA-S levels are much more often measured in studies than DHEA levels, which are often not reported at all. Few published studies have clearly mentioned physiological differences between DHEA to relate the concentration to various diseases, probably due to the different hormone distributions throughout life, compared to its sulfated form (29).

Our findings demonstrated a decrease of HRV in the elderly. In relation to the physiological process of aging, some authors verified that increasing age causes a significant decrease in HRV (30), which is in agreement with our results.

Higher HRV is a signal of good adaptation and characterizes a healthy person, while chronic reductions in resting-state HRV appear to be associated with negative cardiovascular prognostics. A study showed that attenuated HRV is related to a 32%-45% increased risk of cardiovascular events in people who are not known to have cardiovascular problems. However, a 1% increase in the parasympathetic component results in a 1% decrease for risk of fatal and nonfatal cardiovascular disease (31).

We observed that middle-aged individuals and old people aged over 60 years presented a reduced parasympathetic modulation of HR than the young group, which is in agreement with some studies in the literature (32). This result may be related to a lower HRV allied with a higher resting tachycardia. Our analysis focused specifically on a subpopulation of healthy participants without cardiovascular condition and with no reported medication use during the data collection phase. Although the physiological basis of HRV indices is complex, the decreasing HF component of HRV may suggest that aging is accompanied by a reduction of overall fluctuation in cardiac autonomic input and by attenuation in vagal modulation. The changes that occur in parasympathetic modulation as a result of aging may be associated with changes in cholinergic and muscarinic pathways through which the vagal efferent signal is carried. This may include cardiac disorders in acetylcholine release response to stimulation (33), decreases in muscarinic receptor activity (34) and reductions in M2 muscarinic receptor density (35), all of which have been shown to decrease with aging. Loss of vagal reflexes seems to hinder biological functioning and capacity to respond to stimuli originating from the efferent pathway, resulting in increased vulnerability to diseases (9) that are often prevalent in individuals of advanced age (36).

An elevated resting HR, even above 80 beats per minute, was demonstrated to increase the risk of cardiovascular disease (37). Therefore, we elucidated in our study that old men had a higher resting HR than young men, likely to be associated with the hypoactive parasympathetic system found in this study. Our findings are in agreement with the current literature (38). A fast or irregular HR is abnormal and is the result of a problem with the heart or the irregular electrical signals in the heart (39). Knowing resting HR and how it changes over time can provide insight into cardiovascular health. The resting HR increases with age, and it can also be affected by several factors, such as caffeine, tobacco, certain medications and lifestyle. Previous studies have demonstrated that resting tachycardia is a predictor of mortality and shorter life expectancy. These authors estimated that HR increases of 10 bpm are associated with a 20% increased risk of cardiac death, and 15 bpm increases in HR were found to increase the rate of cardiovascular disease mortality (37,39).

In summary, we conclude that low levels of plasma DHEA-S concentration are linked to decreases in cardiac autonomic modulation following increases of age.
